# Risks to human health from ammunition-derived lead in Europe

**DOI:** 10.1007/s13280-019-01194-x

**Published:** 2019-05-16

**Authors:** Rhys E. Green, Deborah J. Pain

**Affiliations:** Department of Zoology, University of Cambridge, David Attenborough Building, Pembroke Street, Cambridge, CB2 3QZ UK

**Keywords:** Bioavailability, Bullet, Gunshot, Health risk, IQ

## Abstract

It has been known for centuries that lead is toxic to humans. Chronic exposure to lead, even at low levels, is associated with an elevated risk of cardiovascular and chronic kidney disease in adults and of impaired neurodevelopment and subsequent cognitive and behavioural development in the foetus and young children. Health agencies throughout the world have moved from assuming that there are tolerable levels of exposure to lead to a recognition that valid ‘no-effect’ thresholds cannot currently be defined. Formerly, the most important exposure pathways were occupational exposure, water from lead plumbing, paints, petrol additives and foods. Regulation of products and improved health and safety procedures at work have left dietary lead as the main remaining pathway of exposure in European countries. Ammunition-derived lead is now a significant cause of dietary lead exposure in groups of people who eat wild game meat frequently. These are mostly hunters, shoot employees and their families, but also some people who choose to eat game for ethical, health or other reasons, and their children. Extrapolation from surveys conducted in the UK and a review of studies of game consumption in other countries suggest that approximately 5 million people in the EU may be high-level consumers of lead-shot game meat and that tens of thousands of children in the EU may be consuming game contaminated with ammunition-derived lead frequently enough to cause significant effects on their cognitive development.

## Introduction

Lead has a wide range of negative effects on human health and functioning. It is not required for essential biochemical functions in any animal species. Adverse effects of exposure to lead on human health occur for most body systems. Some effects occur in individuals with low concentrations of lead in the blood, indicating low levels of exposure (EFSA 2010). In this paper, we first describe the pathway by which humans are exposed to dietary lead derived from spent ammunition. This appears to depend largely upon fragmentation of lead projectiles into small particles when they hit game animals, and absorption from the intestine of dissolved lead from embedded particles when they are ingested along with game meat. We next examine the relationship between the rate at which game meat is eaten by average and high-level consumers and the effects of exposure to dietary lead on the concentration of lead in the blood (B-Pb) and the way in which that is mediated through absorption. Finally, we assess the potential magnitude of effects of dietary exposure to ammunition-derived lead on human health and functioning, the number of people in the EU who may be affected, and the consequences of these effects for society. In taking these last steps, we benefit from the many studies of the relationship between B-Pb, as a measure of exposure to lead from any source, and health outcomes, which are generally accepted to hold regardless of the source of the lead.

## Pathways by which humans are exposed to ammunition-derived lead

### Routes by which lead is absorbed by humans and its fate in the body

Organic forms of lead can be absorbed through the skin, but lead primarily enters the bloodstream following inhalation of contaminated dust and ingestion of contaminated material, including dust, paint fragments, food and water. Previously, lead from paint, lead plumbing and environmental residues of leaded petrol were the predominant routes of non-occupational human exposure to lead. Exposure of humans to lead from these sources has been reduced substantially by increased regulation and substitution of products that contain lead, and reduced occupational exposure through health and safety legislation and its enforcement. The removal of lead additives from vehicle fuel across Europe has resulted in a substantial decrease in lead absorbed through the lungs after inhalation from the atmosphere and by ingestion of dust. Today, most lead exposure of the general population in the EU is from the diet (EFSA 2010). The rate of absorption of ingested lead depends on the age, nutritional status and other individual characteristics and on the physical and chemical characteristics of the lead ingested. Children absorb a larger proportion of the lead they ingest lead than adults (Mushak 1998). After absorption, lead is transported around the body in the bloodstream. It is transferred from the blood to soft tissues such as the liver and kidneys and also to bone where it accumulates. Excretion of lead is via faeces and urine and by incorporation into hair, which is eventually shed. The half-life of lead in blood is about 30 days, but its half-life in bone is several decades (USASTDR 2007). Hence, long-term accumulation of lead, primarily in bone, tends to occur in individuals with regular exposure. As a consequence of accumulation, about 94% of the total lead body burden in adults is in bone, compared with about 73% in children. Lead may be mobilised from bone in times of physiological stress, resulting in elevated blood lead concentrations (USATSDR 2007).

There are several pathways by which people are exposed to ammunition-derived lead. These include (1) via water or plants and animals that have taken up lead derived from spent ammunition deposited in the environment (e.g. review of Green 2015), (2) inhalation by hunters of lead-containing fumes from propellant or lead dust when guns are fired (discussed in Green and Pain 2015a), (3) lead mobilised from lead projectiles and fragments thereof which have been shot into the human body and retained (Weiss et al. 2017), (4) occasional retention of ingested lead projectiles in the caecum, which may cause appendicitis (Clemente et al. 2017), (5) ingestion and absorption of ammunition-derived intracellular lead incorporated into tissues in game meat, although this route is thought to be minor (Green and Pain 2015a), (6) ingestion of lead ammunition or ammunition fragments by eating meat from wild game shot with lead ammunition. In the current review, we focus on the last pathway (6), which may affect a substantial number of people.

### Particles of lead derived from projectiles in meat from game animals killed using lead ammunition

It has long been known that the meat of game animals killed using lead ammunition may contain whole projectiles or large fragments of metal derived from them. However, it was supposed until recently that little of the lead from projectiles shot into game animals was eaten by human consumers because some of the projectiles and their fragments exited the body of the game animal, whilst others were removed during preparation of the meat or detected visually or in the mouth by the consumer and rejected. Because of this supposed detection and removal of almost all ammunition-derived metallic lead from ingested food, it was assumed that only small quantities were ingested and were therefore of little concern. As far as we know, these assumptions have not been made explicit, but we find it difficult otherwise to account for the absence of ingestion of meat from game shot with lead bullets and lead shot as a route of exposure to dietary lead in the Codex Alimentarius Code of Practice on reducing exposure to lead in food (Codex Alimentarius 2004). In addition, these assumptions may account for the absence of a maximum level (ML) for lead in human foodstuffs derived from wild-shot game animals set in the Codex Alimentarius General Standard for Contaminants and Toxins (Codex Alimentarius 2018) and the European Union’s Regulation (EC) No 1881/2006 (EC 2006). MLs were not set for lead in game meat, despite low MLs being set by both systems for many other foodstuffs, including meat from domesticated animals and many wild foodstuffs eaten much less often than meat from domesticated animals, such as cephalopods and bivalve molluscs. This absence of MLs for lead in game meat is surprising, given that various studies described in the following sections have shown that considerable quantities of lead pass into the flesh from the main body of the projectile when it impacts the body of a game animal, some of which is in the form of small particles unlikely to be detected during food preparation or by consumers.

One method of studying this topic is to measure the change in mass of bullets recovered from wild-shot game animals, compared with unfired projectiles. Stokke et al. (2017) retrieved bonded lead core, lead core and copper bullets from carcasses of moose (*Alces alces*). The mean metal loss per bullet on entering the body of the moose was 3.0 g (18–26% of initial mass), 2.6 g (10–25%), and 0.5 g (0–15%) for lead core, bonded lead core, and copper bullets, respectively. Based on hunting and ammunition use data for the year 2013/14, this implies the deposition of 690 kg of lead and 21 kg of copper annually in fragments smaller than the bullets in wild-shot moose carcasses in Finland, Norway and Sweden.

The most frequently used method for detecting projectile fragmentation is X-radiography. Such studies showed that mammals shot using lead bullets often contained lead fragments which were small, numerous and widely dispersed in edible soft tissues away from the wound channels. X-ray studies of red deer (*Cervus elaphus*) (Knott et al. 2010), roe deer (*Capreolus capreolus*) (Knott et al. 2010) and white-tailed deer (*Odocoileus virginianus*) (Hunt et al. 2009; Grund et al. 2010) killed using lead bullets revealed the presence of many small bullet fragments in the edible tissues of the carcass at distances up to 24 cm from the wound channel. Small fragments formed a substantial proportion of the total detected fragment mass (Knott et al. 2010), with 34% of the mass of lead in the eviscerated carcass composed of fragments of 0.01 g mass or smaller (< 1.19 mm diameter). Fragments as small as this have been found not to be completely removed by standard butchery practices used on deer in the USA (Hunt et al. 2009), with at least one bullet fragment being detected in 32% of 0.91 kg packages of minced meat prepared from carcasses of wild-shot deer. Such small fragments in processed meat would probably not be detected by the consumer.

Lead gunshot pellets can also fragment when they are fired into wild game such as gamebirds and waterfowl, leaving small particles detectable on X-radiographs. Small radio-dense metal fragments, identified as shards of metallic lead, were visible on X-radiographs in 76% of 121 wild-shot gamebirds of six species obtained from selected supermarkets, game dealers or game shoots in the UK (Pain et al. 2010). Most fragments were less than about a tenth of a shotgun pellet in size and many were considerably smaller. The small radio-dense particles sometimes appeared to follow the track taken by a shotgun pellet during passage through a bird, were sometimes clustered around bone, but sometimes appeared to be scattered throughout the bird’s body. This study estimated that approximately 0.3% of the mass of lead in the gunshot that struck a gamebird would need to have fragmented into small particles to account for the concentrations of lead subsequently measured by chemical assays of in cooked meals prepared using the gamebird meat. Large fragments of gunshot visible to the naked eye had been removed before preparation of the meals.

Andreotti and Borghesi (2013) X-rayed 196 starlings (*Sturnus vulgaris*) shot in Italy and found lead gunshot pellets and/or visible metallic fragments in 118 carcasses (60.2%). There were abundant tiny fragments embedded in the tissues that would probably be ingested by human consumers. Andreotti et al. (2016) analysed 59 carcasses of woodcock (*Scolopax rusticola*) shot by Italian hunters in Ukraine. 96.6% or carcasses contained ammunition residues and radiographs revealed 215 whole pellets (mean = 3.64 per bird) and 125 clusters of fragments (mean = 2.14 per bird) in 51 and 48 of the birds, respectively. Three quarters of the fragmentation centres contained small particles (< 1 mm diameter).

A limitation of X-radiographic studies of projectile fragmentation is that it has rather coarse resolution. When using two-dimensional X-radiographs, it is difficult to identify images of radio-dense particles as projectile fragments if they are smaller than about 0.1 mm, or even larger. Images of lead particles smaller than this could be confused with bone fragments. In the X-radiographic study by Knott et al. (2010) of metallic fragments in carcasses of deer shot using lead bullets, the smallest fragments identifiable unambiguously were about 0.4 mm in diameter. Ammunition fragments smaller than this might be present but not be detected by conventional X-radiography. A recent study found that many nanoparticles of lead much smaller than this, and of substantial total mass, were present in the meat of animals shot using lead bullets. As well as possibly causing fracture into larger particles, the kinetic energy of a bullet is partly converted, upon striking the target animal’s flesh, into a permanent change in shape of the projectile and into heat. The heat may cause some of the lead to melt or vaporize (Finney et al. 2016). On cooling, very small nanoparticles of solid lead may form from the melt. Kollander et al. (2017) used inductively coupled plasma-mass spectrometry in single-particle mode (spICP-MS) on meat from wild boar (*Sus scrofa*) and roe deer and found large numbers of nanoparticles of lead in samples taken within 10 cm of the wound channel, with no nanoparticles of lead being found in game meat further from the wound channel. The diameter of detected lead nanoparticles was in the range 40 to 750 nm with a median diameter of around 60 nm. Hence, the diameter of even the largest nanoparticles detected by spICP-MS was much smaller than the minimum diameter of lead particles detectable by imaging using X-radiography (100 000 nm). Nanoparticle mass concentration within 10 cm of the wound channel ranged from 290 to 340 ng/g wet weight (290–340 ppb, w.w.), with nanoparticle number concentrations from 27 to 50 million particles per gram of meat. It is possible that metallic nanoparticles might be absorbed unmodified through the wall of the intestine or into epithelial cells or that their high surface:volume ratio could result in much of the nanoparticle lead being dissolved and absorbed as lead salts. The substantial gap between the largest nanoparticles of lead detected by spICP-MS and the smallest fragments detected by X-radiography raises the question of whether there are also lead particles in game meat intermediate in size between those detected by the two methods. Nanoparticles of lead have only been searched for so far in game animals killed using lead bullets. Gunshot pellets seem less likely to give rise to nanoparticles of lead because of their lower impact velocity, but whether such nanoparticles are absent from animals killed using lead gunshot remains to be established.

### Concentrations of lead in the meat of game animals consumed by humans

Many chemical analyses of the concentration of lead in the tissues of game animals killed using lead ammunition have been published. However, it is not always clear what the significance of these concentrations is for human health because some analyses include lead from whole gunshot and large projectile fragments present in meat samples, which would almost certainly be removed during food preparation or by the consumer (see preceding section). For example, EFSA (2010) reviewed a large number of determinations of lead concentration in game meat, but it was unclear for most of them whether whole shot and/or large projectile fragments had been removed or not. Hence, in this paper, we concentrate on recent studies in which protocols regarding removal of projectiles, large fragments and wound channel tissue were clearly defined or meat already processed for human consumption was analysed. We also summarise published reviews of the older literature.

Dobrowolska and Melosik (2008) measured lead concentrations in samples of muscle tissue from ten wild boar and ten red deer shot with lead bullets. Lead concentrations in muscle tissue were elevated above the background level at up to 30 cm from the bullet channel. Butchering and food preparation procedures on these boar and deer would have required that a substantial proportion of muscle be discarded if all tissue retained for human consumption was to have lead concentration within the ML set by the EU of 100 ppb (w.w) for non-game meat (bovine animals, sheep, pig and poultry—excluding offal). Lindboe et al. (2012) found that the mean concentration of lead in random samples of ground meat from moose killed in Norway with lead-based bullets was 5600 ppb (w.w). Danieli et al. (2012) analysed lead levels in muscle and liver taken from 54 wild boar shot in central Italy. Areas damaged by the bullet were discarded and all meat was taken from more than 40 cm from tissue damage or the bullet tract. Mean lead concentrations were 124 ppb (w.w.) in muscle and 329 ppb in liver.

Ertl et al. (2016) analysed concentrations of lead in muscle tissue from pheasants (*Phasianus colchicus*) and five wild mammal species shot in Austria. Gunshot pellets and wound channel tissue were excluded from the samples taken for analysis. Mean lead concentrations were 77 000 ppb (w.w.) in muscle from chamois (*Rupicapra rupicapra*), 9000 ppb (w.w.) in meat from brown hares (*Lepus europaeus*), 125 000 ppb (w.w.) in pheasant, 6 ppb (w.w.) in red deer, 140 ppb (w.w.) in roe deer and 15 ppb (w.w.) in wild boar. Animals killed using gunshot pellets had particularly high lead concentrations. Lehel et al. (2016) found that the mean lead concentration in muscle samples unlikely to contain large bullet fragments taken from roe deer killed in Hungary using lead bullets was 480 ppb (w.w.). Vogt and Tysnes (2015) collected a sample of packs of minced meat from wild-shot moose from 27 groceries in southern Norway. The mean lead concentration was 1790 ppb (w.w.). Only 27% of packs had a lead concentration below 100 ppb (w.w.), the EU’s ML for non-game meats.

Johansen et al. (2004) found that lead contamination of the meat of seabirds killed using lead shot occurred even though shot was removed after cooking. Pain et al. (2010) found a mean lead concentration of 1181 ppb (w.w) in meals prepared from 121 wild-shot UK gamebirds of six species, from which shot and large fragments had been removed prior to meal preparation and analysis, with no significant variation among species. Lead concentrations in the meals were correlated with the number of shotgun pellets and large fragments of lead removed before chemical analysis but also, after this effect was allowed for statistically, with the number of small radio-dense fragments detected by X-radiography. These small fragments could not easily have been removed. High concentrations of lead occurred in some meals prepared from birds in which no whole pellets or large fragments at all were apparent on X-rays. The only plausible mechanism for this observation is that lead particles remained in the meat after the removal of whole shot and large fragments, conforming to usual culinary practice. McAuley et al. (2018) reported lead concentrations in meat from grouse (*Bonasa* and *Falcipennis* sp.) killed in Canada using lead ammunition (mostly shotgun pellets). Pellets and projectile fragments were removed prior to analysis. Grouse breasts (two per bird) were categorised as having visible signs of shot impacts on the exterior or not and the two classes were analysed separately. The mean concentration of lead in impacted breast muscle was 968 ppb (w.w.), with the concentration in non-impacted breasts being 13 ppb (w.w.).

The results presented above show that mean lead concentrations in the meat of wild game mammals and birds shot with lead ammunition and eaten by humans are often one or two orders of magnitude higher than the non-game meat ML of 100 ppb (w.w.) set by the European Commission, even when projectiles, large fragments and wound channel tissues were discarded. In the next section, we examine evidence that lead present in ingested game meat is absorbed, resulting in consumers having higher B-Pb than would otherwise be the case.

### Potential effects of improved meat preparation practices on concentrations of lead in the meat of game animals

Several examples are given in the previous section of normal food preparation methods leaving elevated concentrations of lead in the meat of large game animals and gamebirds. This section examines the practicality of decreasing lead concentrations in meat consumed by humans by more stringent butchery practices. Lead concentrations in the meat of large game animals are usually much higher within 30 cm of the wound channel than further away (e.g. Dobrowolska and Melosik 2008). Butchering to remove meat within a 30 cm radius around the wound channel left by a lead bullet would, therefore, be expected to remove much of the ammunition-derived lead, but it is not a usual practice to discard such a large proportion of meat because this is seen to be wasting a large amount of food. For small game, such as gamebirds, it would be impractical to remove all of the meat potentially contaminated with lead from gunshot as multiple gunshot usually enter a small game animal and fragments can be widely dispersed (Pain et al. 2010). The same difficulty arises for the preparation of meat from wild-shot brown hares (Stankevičiūtė et al. 2013). Kollander et al. (2014), reported a study of enhanced meat preparation in which twenty crows (*Corvus corone*) were shot, ten with each of two shot types: a sports shooting shot sometimes used for small game, and a standard small game shooting shot. Breast muscle was removed, damaged tissue separated and shot removed prior to analysis. The sports shooting shot type resulted in mean tissue lead levels almost 400 times the EU ML before cleaning, falling to 14 times the EU ML after cleaning. The game shooting shot type resulted in mean tissue Pb levels 1840 times the EU ML before cleaning (as one shot remained in a sample; 1.8 times the EU ML of 100 ppb excluding this sample), dropping to the EU ML, following cleaning. This small study shows that, even with careful butchering, mean lead levels following cleaning did not drop below the EU ML for other meats. In addition, crows are different in structure and size from gamebirds and waterfowl and are not widely eaten by humans. Hence, they may not be an appropriate model species for a study of the effects of meat preparation methods for small game in general. A study of 121 gamebirds from the UK found mean lead concentrations in edible meat of 1181 ppb (12 times the EU ML) after whole shot or large (visible) fragments had been removed, being some 6.6 times higher than that of the 9 crows (if the outlier containing a shot is excluded) in the Kollander et al. (2014) study. Of the five bird species surveyed by Pain et al. (2010) that could legally be shot with lead in the UK, the lowest mean concentration of lead found in meals prepared from the meat was 433 ppb for woodpigeon (*Columba palumbus*). This lowest mean still greatly exceeds (by a factor of 2.4 times) the lead concentration in meat in the crows shot with typical game shooting ammunition, before cleaning (Kollander et al. 2014).

Strict protocols to dispose of the damaged meat removed by enhanced butchery and prevent it from entering the human food chain would need to be established if consumers were to be confident that it would not be incorporated into other products destined for human consumption, such as game sausages or minced meat, or included in food for other animals, such as domestic dogs. We conclude that lead concentrations in game meat eaten by humans could be reduced by discarding a higher proportion of meat from carcasses than is the current usual practice, but the amounts required to be discarded might be substantial and make practical implementation difficult to achieve and verify, especially for small game animals.

### Bioavailability of ammunition-derived lead present in game meat and the effect of its ingestion on the blood lead concentration of consumers

We know of no experimental studies of humans to measure the bioavailability of dietary lead derived specifically from ingested ammunition residues. However, Hunt et al. (2009) fed domesticated pigs (*Sus scrofa domesticus*) on two meals, separated by 1 day, of minced meat from deer shot with lead-based bullets, which was known from X-radiography to contain small bullet fragments. The pigs’ B-Pb was then measured and compared with that of controls fed on deer meat that contained no fragments. Two days after first ingestion of fragment-containing venison, mean B-Pb of the treated pigs peaked at 2.29 μg/dL, which was 3.6 times higher than that of controls (0.63 μg/dL). B-Pb levels in the experimental pigs returned to being similar to those of controls within 6 days of the last ingestion of contaminated meat. Isotope ratios of lead in the meat matched those of the lead in the bullets used to shoot the deer, supporting the contention that the absorption by the pigs was of dietary lead derived from the ammunition. The quantity of ammunition-derived lead ingested by the pigs was not measured, so this experiment showed that ammunition-derived dietary lead is bioavailable to pigs, but did not measure its absolute bioavailability.

Several comparative studies indicate that the B-Pb of people who eat game animals killed using lead ammunition is elevated compared to that of people who do not consume game meat and some of these show that the degree of elevation of B-Pb is related to the amount of game meat eaten. These studies indicate that some ingested ammunition-derived lead is absorbed (Dewailly et al. 2001; Bjerregaard et al. 2004; Johansen et al. 2006; Iqbal et al. 2009; Bjermo et al. 2013; Meltzer et al. 2013; Knutsen et al. 2015). In addition, analysis of stable isotope ratios of lead in blood samples indicates that exposure to ammunition-derived lead is the main cause of elevated blood lead (B-Pb) in indigenous people in Canada (Tsuji et al. 2008). Knutsen et al. (2015) reviewed several studies of the effect of game meat consumption on B-Pb including: (1) a 2012 assessment by the Norwegian Scientific Committee for Food Safety (VKM) of lead exposure from cervid meat to the Norwegian population and (2) a Norwegian Institute of Public Health investigation of associations between cervid meat consumption and B-Pb in Norwegians (the Norwegian Game and Lead study). There was an increase of approximately 31% in blood lead concentration associated with cervid game meat consumption once a month or more, mostly associated with consumption of minced cervid meat. A few studies have reported no significant effect of eating game meat on B-Pb, but they have significant weaknesses. Haldimann et al. (2002) did not find elevation of B-Pb associated with intake of lead from game meat when they compared B-Pb of 31 hunters with that of 42 controls. However, no information was available for the anonymous control group beyond age and sex. Whether or not they were active hunters, their level of game consumption and other variables that might affect exposure to lead were unknown. In addition, the effects of potentially confounding variables, such as drinking and smoking habits, did not appear to have been taken into account in the statistical analysis. With this small sample size and the lack of adequate controls we, therefore, consider a conclusion of no effect to be unreliable. Fustinoni et al. (2017) measured B-Pb in 70 consumers of game meat and 25 controls and concluded that game meat consumers did not have higher levels of B-Pb when other variables, namely hunting and wine drinking, were taken into account statistically. Mean B-Pb was about twice as high for game meat consumers as it was for non-consumers, but the authors argued that this was because of confounding effects of the other variables. We consider this conclusion to be unreliable because people who had consumed game within the week before sampling were excluded from the study. Given the experimental finding of Hunt et al. (2009) that the elevated B-Pb of pigs fed on ammunition-derived lead had returned to normal within 6 days of last eating contaminated meat, this exclusion would probably bias the measured B-Pb of frequent game meat consumers to be lower than their usual level, as well as excluding some high-level, very frequent consumers of game from the study. In addition, the ‘frequency of hunting’ variable included in the multiple regression analysis was positively correlated with the ‘game meat consumption’ variable. This correlation between explanatory variables makes conclusions about the relative importance of the two variables problematic.

Taken together, the findings summarised above indicate that B-Pb of humans tends to increase in association with consumption of game meat containing ammunition-derived lead due to absorption of ammunition-derived lead from the alimentary canal, as was demonstrated experimentally for pigs by Hunt et al. (2009). However, without further analysis, these observations do not indicate what proportion of the ammunition-derived lead ingested is absorbed or how much B-Pb is increased per unit of dietary lead ingested. In the absence of experimental data, such estimates require correlational studies in which both the intake of lead and the elevation of B-Pb are measured.

Green and Pain (2012) used observations from two studies of Greenland adults (Bjerregaard et al. 2004; Johansen et al. 2004, 2006) to derive a quantitative empirical relationship by linear regression modelling between the mean daily intake of dietary lead from the meat of birds killed using lead shot and the mean concentration of B-Pb. There was a strong relationship in the data from both Greenland studies between mean B-Pb and the estimated mean rate of intake of dietary lead from meals of cooked wild bird meat. The regression models of Green and Pain (2012) indicated that the effect of ingested ammunition-derived lead on B-Pb was 39% lower than that expected for lead not derived from ammunition (Carlisle and Wade 1992). The absolute bioavailability of dietary lead derived from ammunition (the proportion of the ingested amount which is absorbed and enters the blood) might be expected to be lower than that of lead in the general diet because some of the ingested ammunition lead may remain as metallic fragments after cooking and processing in the alimentary tract. Metallic lead, especially that remaining in large fragments, may not be totally dissolved nor be absorbed in the intestine as readily as more soluble lead salts and complexes (Barltrop and Meek 1975; Oomen et al. 2003). However, it should also be noted that this regression method is subject to a well-known bias. Least squares regression assumes that the independent variable (in this case the dietary lead intake rate) is known without error. This is not the case because the intake rate means used were determined from sample estimates with attached errors which cannot be fully quantified and adjusted for. The direction of this bias on the slope of the fitted regression is negative, meaning that the true absolute bioavailability of lead may be larger than that estimated by this method.

There appear to be no published studies in which B-Pb was related to ingestion rates of ammunition-derived lead in children. The bioavailability of lead in the ordinary diet is considerably higher in children than in adults (Mushak 1998; IEUBK 2010). Green and Pain (2012) assumed that the ratio of the absolute bioavailability of dietary lead from cooked wild bird meat to that of lead from the ordinary diet, calculated for adults (above), would be the same in children. As there is a widely used value for the absolute bioavailability to children of lead from the ordinary diet (0.5, from Mushak 1998; IEUBK 2010), they estimated a value for absolute bioavailability in children of dietary lead derived from the cooked meat of wild birds of 0.3060. The same caveat about probable negative bias in this estimate applies as that described above for adults.

We conclude that a considerable proportion of the ammunition-derived lead present in food ingested by consumers is absorbed into their blood and that this results in elevated B-Pb of game consumers, probably for a period of days after ingestion. This lead then moves into other tissues or is excreted.

## Effects of lead on human health and functioning

### Principal effects of lead on human health

Once lead has been absorbed into the body, its effects on health and functioning are thought to be independent of its original source. Hence, correlations between health outcomes and concentration of lead in tissues are an important source of information on effects of lead on health. The concentration of lead in whole blood (B-Pb) is the most widely used measure of recent exposure, because of the short half-life of lead in the blood. Although measurements of lead concentrations in other tissues, such as teeth and bone, might be more informative about long-term exposure and chronic effects on health, large-scale sampling of them has seldom been achieved. However, Needleman et al. (1979) measured long-term exposure of children to lead by analysing teeth, and the recent use of X-ray fluorescence for measuring bone lead may also make such studies more practical (Specht et al. 2019). Hence, much of what is known about the health effects of lead is based upon correlations between health outcomes and B-Pb.

The consequences for human health of exposure to lead from many sources have been considered in great detail by the appropriate authorities of several countries. Lead affects the nervous, urinary, cardiovascular, immune, reproductive and other body systems and a range of organs, including the brain (USATSDR 2007; EFSA 2010). Experiments show that high doses of lead can induce tumours in rodents, and possibly humans, and the International Agency for Research on Cancer classified inorganic lead as ‘probably carcinogenic to humans’ (Group 2A) in 2006 (IARC 2006). Body systems particularly sensitive to low levels of exposure to lead include the hematopoietic, nervous, cardiovascular and renal systems (EFSA 2010).

Green and Pain (2012) used a statistical model fitted by Borja-Aburto et al. (1999) to describe the relationship between B-Pb and the proportion of pregnant women in Mexico City who incurred spontaneous abortion. The model adjusted for the effect of a previous history of spontaneous abortion. EFSA (2010) did not evaluate this study, which indicated an increased risk of spontaneous abortion for women with high B-Pb levels.

The effects of lead on the developing nervous system appear to persist to influence the academic performance of children of school age. In a study published too late to be assessed by the EFSA CONTAM Panel, Chandramouli et al. (2009) reported a marked negative association of academic test results of UK schoolchildren at Key Stage 1 (SATs tests) with B-Pb measured at 30 months of age. To define a change in SATs KS1 writing score equivalent to that of 1 IQ point, identified as the BMR for IQ by EFSA (2010), Green and Pain (2015b) first obtained the maximum-likelihood mean and standard deviation of SATs scores for children in England in 2010 (Department for Education 2013). They then divided this value by 15 (because the standard deviation of population IQ is 15) to obtain the BMR for the SATs KS1 writing grade score. They estimated that, in a typical year, thousands to tens of thousands of children in the UK are consuming sufficient quantities of game shot with lead ammunition for the effect to exceed this BMR level.

Persistent effects of lead on cognitive function over even longer periods were reported by Reuben et al. (2017) in New Zealand. They related IQ measured at age 38 years to B-Pb measured at 11 years. After adjusting statistically for effects of maternal IQ, childhood IQ, and childhood socioeconomic status, each 5-µg/dL increment in B-Pb in childhood was associated with a reduction in IQ at 38 years old of 1.61 points. Thus, they found not just an association between greater blood lead levels in childhood and adult IQ, but also with a decline in IQ from childhood to adulthood.

### Changes in approach to the evaluation of risks from chronic low-level exposure to lead

In recent decades, public health authorities have made fundamental changes in their approach to the identification of tolerable rates of exposure to dietary lead. Up to the 1990s, it was assumed that there were levels of exposure to lead that would not give rise to significant adverse effects on health, but this was followed by increasing recognition that thresholds below which exposure was safe could not be determined. These developments are summarised in Table 1.

**Table 1:** Evidence-based changes in the approach to the evaluation of risks from chronic low-level exposure to lead

Event	References
Public health authorities formerly identified a tolerable rate of dietary intake of lead intended to maintain exposure below an assumed no-observed-adverse-effect-level (NOAEL). An example is the Provisional Tolerable Weekly Intake (PTWI) of lead for infants and children set by the World Health Organization Joint Expert Committee on Food Additives and Contaminants (JECFA) in 1982. The PTWI approach was endorsed by the EU Commission’s Scientific Committee on Food. In the EU, this approach, together with data on lead exposure, resulted in the setting of Maximum Levels of lead in many foodstuffs in the EU Regulation (EC) No 1881/2006	SCF (1994), SCOOP (2004)
The U.S. Environmental Protection Agency, California EPA and World Health Organization concluded that lead is a substance for which a threshold level for negative effects on human health cannot currently be determined. This rendered the NOAEL and PTWI approaches suspect	USEPA (2006), CalEPA (1997, 2009), WHO (2009)
The European Commission requested the European Food Safety Authority (EFSA) to produce a scientific opinion on the risks to human health related to the presence of lead in foodstuffs including to consider whether the PTWI of 25 μg/kg b.w. was still appropriate	EFSA (2010)
The EFSA CONTAM Panel identified developmental neurotoxicity in young children and cardiovascular effects and nephrotoxicity in adults as the critical effects for the risk assessment	EFSA (2010)
A meta-analysis of the results of seven studies published between 1989 and 2003 of the IQ of 1333 children in relation to B-Pb, and a refinement/reanalysis of the same data found marked decreases in IQ with increasing B-Pb, even at low B-Pb values.	Lanphear et al. (2005), Budtz-Jørgensen (2010), EFSA (2010)
Meta-analyses supported a relatively weak, but statistically significant, association between B-Pb levels and systolic blood pressure, amounting to an increase in systolic blood pressure of approximately 1 mmHg with each doubling of B-Pb without any clearly identifiable B-Pb threshold for this effect	Staessen et al. (1994), Nawrot et al. (2002) EFSA (2010)
A range of cross-sectional and prospective longitudinal studies were conducted to examine the relationship between serum creatinine levels, which rise when kidney filtration is deficient, and B-Pb. Studies suggest an increased likelihood of chronic kidney disease as B-Pb levels rise. EFSA CONTAM Panel concluded that nephrotoxic effects are real, that they may be greater in men than women and that they are exacerbated by concurrent diabetes or hypertension	EFSA (2010)
EFSA (2010) concluded that there is no evidence for a minimum B-Pb threshold below which effects on IQ, systolic blood pressure and chronic kidney disease do not occur. Hence, they considered that the NOAEL and PTWI approaches were not supported by evidence	EFSA (2010)
Abandonment of NOAEL and PTWI approaches by EFSA was followed by similar conclusions by the WHO/FAO JECFA, Health Canada, and the Centers for Disease Control and Prevention in the United States	JECFA (2011), Health Canada (2012), ACCLPP (2012)

Based on an analysis of the extensive existing information, EFSA (2010) proposed that approaches that assumed the existence of safe thresholds should be replaced by the use of the Benchmark Dose (BMD) approach. The BMD is the B-Pb concentration associated with a pre-specified change in response (i.e. a specified loss of IQ, increase in systolic blood pressure, increased incidence of chronic kidney disease), the Benchmark Response (BMR). EFSA (2010) proposed BMRs that could have significant consequences for human health on a population basis as a one point (1%) reduction in IQ, a 1% increase in systolic blood pressure (SBP) (equivalent to a 1.2 mmHg change), and a 10% increase in expected incidence of chronic kidney disease (CKD) as the BMR for nephrotoxicity (EFSA 2010). EFSA (2010) also proposed that significant levels of exposure should be set using Benchmark Dose Limit (BMDL) reference points, which take into account statistical uncertainty in the determination of BMDs.

Although relevant agencies have determined that there are no currently defined thresholds for lead intake below which there are expected to be no negative effects on health, there remains a tendency to treat some exposure levels below those expected to cause the BMR as “essentially negligible”. However, as Wilson and Richardson (2013) comment, this “is a matter of regulatory policy, not science”. In our view, the implied acceptance of avoidable and quantifiable negative effects on health is undesirable.

## Hazards to human health from ammunition-derived dietary lead in europe

EFSA (2010) used information on lead concentrations in food and amounts of food eaten by individuals in 19 participating countries to calculate mean (‘average base diet’) and 95th percentile (‘high base diet’) lead dietary exposures separately for each country. These data were presented as ranges, from the country with the lowest average exposure to that with the highest average exposure. These exposure data were then used to estimate corresponding B-Pb concentrations, and these were compared with Bench Mark Doses, adjusted for uncertainty (BMDLs) to evaluate risk (Table 2). Adults, but not children, frequently consuming game meat (defined as one 200 g meal per week of game) were considered separately for average base diet consumers, but not for high base diet consumers. In calculating the effects upon B-Pb of game meat consumption the EFSA (2010) assumed that the bioavailability of dietary lead directly derived from ammunition was the same as for other sources of dietary lead. They obtained the ratio of dietary exposure, assuming various diets, to the BMDLs. The risk of Benchmark Responses occurring was considered to be of particular concern if this ratio exceeded one. EFSA (2010) concluded that there was a potential risk that some children in groups with average and high base diets could incur reductions of one IQ point as a result of exposure to dietary lead. Exposure to additional lead from frequent consumption of game, while not specifically evaluated, would further increase this risk in those exposed. EFSA (2010) concluded that risk of cardiovascular effects as a result of exposure to dietary lead was very low for adult average consumers across European countries. However, if exposure to dietary lead was closer to the upper end of the range in adult high consumers, the potential exists for some consumers to have increased systolic blood pressure as a result of exposure to lead. For nephrotoxicity, EFSA (2010) concluded that it is possible some consumers of an average base diet at the high end of the exposure range, and of consumers of a high base diet across the exposure ranges could potentially incur chronic kidney disease as a result of exposure to dietary lead. For consumers of an average base-diet, but also with frequent consumption of game meat, EFSA (2010) concluded that there was a risk that some people could incur cardiovascular and nephrotoxic effects as a result of exposure to lead. In summary, EFSA (2010) concluded that, at current levels of dietary lead exposure, there is only a low to negligible risk of clinically important effects on either the cardiovascular system or kidneys of adult consumers, for the population in general. However, the results indicate that groups of adults who are frequent consumers of game are at increased risk of suffering clinically important effects. There is also concern about possible effects on neurodevelopment at current levels of exposure to lead for infants, children and pregnant women. The effects of frequent consumption of game shot with lead ammunition was not specifically studied for these most vulnerable groups, but would be expected to further increase exposure and risk.

**Table 2:** Daily dietary lead intake values across the EU and lead intake values corresponding to blood lead levels associated with Benchmark Dose Limit reference points for effects on IQ, Systolic Blood Pressure and Chronic Kidney Disease. Taken from EFSA (2010)

BMDL	Blood lead (µg/L)	Lead intake (µg/kg b.w./d)
IQ—BMDL_01_	12	0.50
SBP—BMDL_01_	36	1.50
CKD—BMDL_10_	15	0.63
Daily lead intake across EU participating countries µg/kg b.w./d		
Adults, average base diet		0.36–1.24
Adults, average base diet with game		1.98–2.44
Adults, high base diet		0.73–2.43
Children, average base diet		0.80–3.10
Children, high base diet		1.71–5.51

The BMDL is the 95th percentile lower confidence limit of the benchmark dose (BMD) of 1% extra risk (BMDL_01_) used as a reference point for the risk characterisation of lead when assessing the risk of intellectual deficits in children measured by the Full Scale IQ score (1 IQ point reduction) or a 1% increase in systolic blood pressure (SBP). For chronic kidney disease, the BMDL_10_ represents a 10% increase in prevalence

Green and Pain (2015b) estimated numbers of people in the UK in at-risk groups expected to have intake rates of ammunition-derived dietary lead that would result in effects exceeding the EFSA (2010) BMR levels. They used data on lead concentrations in UK gamebirds, from which gunshot had been removed following cooking to simulate human exposure to lead (Pain et al. 2010), in combination with UK national diet survey data and surveys of numbers of high-level consumers of game meat and their levels of consumption. They found that at least one million people in the UK consume wild game at least once per year and surveys indicate that at least tens of thousands of people from the shooting community are high-level consumers of wild-shot game. The mean frequency of consumption of game meat by these high-level consumers may exceed one game meat meal per week, averaged over a whole year. It was estimated that thousands of children in the UK (calculated to be in the range 4000–48 000) were at potential risk of incurring a one point or more reduction in IQ as a result of current levels of exposure to ammunition-derived dietary lead. Numbers of adults potentially vulnerable to critical health effects appear to be smaller, but the available data are too sparse to be certain.

No detailed studies have been conducted across the EU or Europe to evaluate the numbers of children or adults at risk of negative health effects caused by ingested lead ammunition, but an approximate indication of possible numbers can be obtained. EFSA defined ‘high-level consumers’ of game meat as adults who eat an average of at least one 200 g meal of game meat per week. Weights of meals vary as will lead exposure depending upon the type of meal (gamebird or large game). While the amount of wild game consumed in all EU countries is not known, Pain et al. (2019), proposed an approximate estimate for children by scaling the number of UK children exposed to high dietary levels of ammunition-derived lead (c. 10 000) by the number of hunters in other EU countries, relative to numbers in the UK. The main assumption of this approach is that overall per capita game consumption by hunters and their families is fairly consistent across EU countries and is similar, on average, to the level found in the UK. Based on these assumptions, it was estimated that 83 000 or more children across the EU27 may be at risk of a reduction in IQ of 1 point.

Green and Pain (2015b) also used UK National Diet and Nutrition Survey (NDNS) data to estimate the total number of people estimated to consume gamebird meat in a typical 4-day period, averaged over the whole year. This was 2.52% of the UK population (95% CL 2.02–3.01%). This rate may be representative of the situation at any time of year as proportions of people eating gamebird meat appear to be similar within and outside the hunting season (Taylor et al. 2014). To consider an extreme assumption: if the same people eat gamebird meals consistently in every 4-day period throughout the year then over 2.52% of the population would eat at least one gamebird meal per week, averaged across the year. At the opposite extreme, if people who eat gamebird meat only do so occasionally, then a higher proportion of the population will eat gamebirds but with a lower frequency. The most extreme case of this kind that would yield the same mean rate of gamebird consumption per 4-day period observed in the diet survey would be if 90.3% of the UK population ate just one gamebird meal per year. However, we can be sure that this hypothetical uniform low level of game consumption is not the case. A survey of members of the shooting community in the UK, conducted by the British Association for Shooting and Conservation (BASC) and the Countryside Alliance (CA), estimated that about 9000 (midpoint of the range 5500–12 500) children under 8 years old and about 44 500 adults (midpoint of the range 27 000–62 000) from that community consume at least one game meal per week, averaged over the whole year, with all types of game being included (cited in LAG 2014). These 53 500 adults and children from the shooting community represent 0.084% of the UK population. The percentage of high level consumers of game in the UK would, therefore, appear to lie between 0.084% and 2.52% of the population.

In Italy, Ferri et al. (2017) used questionnaire surveys to study game consumption by 766 Italian shooters. An average of 100–200 g game per serving was consumed with an average of four servings per month (once per week). The highest rate of game intake recorded was 3 kg per month, and game was regularly consumed with friends and relatives. From these results, it was estimated that 3% of the Italian population were regular consumers of wild game. Danieli et al. (2012) found that hunters and their families in central Italy (221 adults and 41 children) regularly ate wild boar throughout the year with mean weekly consumption of 123 g meat, 45 g liver by adults, and 49 g meat, 10 g liver by children. 80 adults and 18 children ate both meat and liver.

In Spain, 6.3% of 3000 people surveyed reported eating game meat of all kinds (large game, partridge, quail or rabbit), but the frequency of consumption was not given (AESAN 2012). In a separate study of people that reported eating game, Sevillano Morales et al. (2011) found that average consumption of red deer and wild boar, in people that consumed both meats, was 8.4 kg/year (maximum 56.67 kg/year) for hunters (*n* = 90) and 4.41 (max. 55 kg/year) in non-hunters (*n* = 50). Maximum consumption was by two people who only ate large game meat, so represents an outlying extreme. Sevillano Morales et al. (2018) found mean game meat consumption of 8.57 kg/person/year (of wild boar, red deer, rabbit (*Oryctolagus cuniculus*), partridge (*Alectoris rufa*) and processed meat products made from them) in 337 habitual consumers of game (hunters and their relatives).

In Germany, Gerofke et al. (2018) conducted a game consumption survey of a representative sample of 1000 people and found that the minimum proportion of people that consumed meals of at least one of either red deer, roe deer and wild boar was: more than once per week (0.8%); once per week (0.7%); one to 3 times per month (2.4%); 6–11 times per year (2.3%); one to 5 times per year (31.9%). Game consumption figures for the three species were given separately and the authors do not state whether those people that ate one species also ate other species. Hence these rates are minima.

No reliable data exist on the amounts of game consumed in France, but ANSES (2018) performed a risk assessment in which most consumers of game were considered to be hunters and their relatives. Since 1.1 million people have a hunting permit, a total of about 3–4 million people were thought to consume game. This comprises about 6% of the population. Among these, two scenarios of game consumption were considered, those of regular (e.g. 200 g every 2 weeks) or high (200 g per week) consumption.

Sweden has an annual “production” of game meat corresponding to approximately 12% of the production of beef, and about 600 000–900 000 persons (7–10% of the population) were estimated to be potential high consumers of game, though there was no definition of a high rate of consumption (Ankarberg et al. 2013).

Taken as a whole, the studies described above indicate that the main consumers of game are hunters and their families and associates, and that a few percent of the population may be frequent (a few times per month) or high (once per week or more) consumers of game in most countries. While we have only been able to find any kind of estimate or assumed consumption levels for six countries, these countries hold more than two-thirds (67%) of all EU hunters (FACE 2010) and include the five countries with the most hunters, i.e. France, Spain, UK, Italy and Germany. Consequently they are likely to be broadly representative of the EU and illustrate that the number of people at potential risk of health effects from lead in game is non-negligible across the EU. Taking the consumption of at least one meal of game meat per week, averaged across the whole year, as the definition of a high-level game consumer, and assuming that 1% of the total population of the EU countries are high-level consumers, gives a rough estimate of about 5 million high-level consumers in the EU.

In accord with these conclusions, the UK Food Standards Agency (FSA 2012) have advised that frequent consumers of game shot with lead ammunition should eat less of this type of meat, and that this is especially important in the case of toddlers and children, pregnant women and women trying for a baby, because of the harm that lead can cause to the brain and developing nervous system. The FSA does not recommend any lower level of consumption as being safe for these at-risk groups. This is similar to recent advice given following risk assessments by equivalent agencies in a range of other European countries who consider that these most vulnerable groups should eat little or no game shot with lead ammunition (AESAN 2012; Knutsen et al. 2015; ANSES 2018; Gerofke et al. 2018). The risks to vulnerable groups such as frequent consumers, women of childbearing age especially who are pregnant or trying to become pregnant, and children from eating game shot with lead, are acknowledged by FACE, the European Federation for Hunting and Conservation, which is the international organisation set up in 1977 to represent the interests of European game hunters. FACE recommend that women who are pregnant or planning to get pregnant and children < 7 years should avoid eating lead-contaminated meat (FACE 2018). FACE suggest that these groups can reduce their risk by eating game killed using non-lead ammunition, removing lead fragments by sufficient cleaning or consuming less game. However, consumption of less game shot using lead ammunition does not constitute the avoidance that FACE recommend. For reasons we explain in an earlier section, we are also sceptical, from the evidence available so far, that practical methods for preparing the meat of small game would achieve the avoidance of ingestion of lead-contaminated meat. We suggest that avoidance of any game, avoidance of game shot with lead ammunition, and only consuming game killed using non-lead ammunition are the practical methods by which at-risk groups can achieve the avoidance of lead-contaminated meat recommended by FACE.

While our review focuses on risks from exposure to ammunition lead in Europe, these risks are present anywhere where people frequently consume wild game shot with lead ammunition. Game consumption levels may vary between countries, and include everything from bushmeat harvested with lead (e.g. in Benin, Ahmadi et al. 2018) to extreme cases where individuals subsist almost exclusively on self-killed game (e.g. a case study of a hunter in New Zealand, Buenz and Parry 2017).

## Wider consequences of ammunition-derived dietary lead for human health and well-being

As more information becomes available from long-term studies, there is increasing evidence of effects on human health and well-being of low-level environmental lead exposure, and these have implications for exposure from any source. For example, Lanphear et al. (2018), in a study of > 14 000 adults, found that an increase in B-Pb from 1.0 to 6.7 μg/dL was associated with mortality from cardiovascular disease and ischaemic heart disease. The authors concluded that low-level lead exposure is an important, and largely overlooked, risk factor for cardiovascular disease mortality in the USA.

In addition to the health effects and lack of attainment probably experienced by individuals as a result of frequent exposure to ammunition-derived dietary lead, there are likely to be consequences for societies which are felt more widely. Additional ill-health caused by dietary lead results in additional costs and burdens for health services and taxpayers. More difficult to assess are effects on society mediated through effects of exposure to lead on human behaviour, including antisocial and criminal behaviour. In the twentieth century, there were large changes in exposure of humans to environmental lead caused by the introduction of regulations to reduce or prevent the use of lead in products, especially leaded petrol. These regulations differed geographically in timing and scope, thus constituting a set of uncontrolled natural experiments on the effects of changes in exposure to environmental lead on human behaviour. Nevin (2007) reported an association between preschool blood lead levels in the USA, Britain, Canada, France, Australia, Finland, Italy, West Germany, and New Zealand and subsequent trends in rates of crime, arrest and incarceration. These observations were most consistent with effects of exposure to lead causing neurobehavioral damage in the first year of life. Reyes (2015) analysed the association of early childhood lead exposure with antisocial behaviour problems from childhood into early adulthood for two cohorts of children in the USA and found marked effects. A prospective longitudinal study found significant relationships between prenatal and postnatal exposure of children to lead, determined by blood sampling, and covariate-adjusted rates of self- and parent-reported antisocial and delinquent behaviours later in life (Dietrich et al. 2001). Boutwell et al. (2017) conducted a correlational study in St Louis, USA, in which they examined the association of geographical variation among regions of the city (called ‘census tracts’) in lead exposure (from B-Pb measurements) and various indicators of criminal and antisocial behaviour. The study B-Pb levels were statistically significant predictors of the incidence of firearm crimes, assault, robbery and homicide, even after accounting for important sociological variables. However, they found no significant association between B-Pb and the incidence of rape.

At a population level, the differences and changes in B-Pb examined in these studies of the links between lead exposure and crime, which resulted largely from differences and changes in exposure to leaded petrol, were large compared with those expected to be caused by reducing exposure to ammunition-derived lead. Nonetheless, the possible consequences for groups currently exposed to this toxic dietary contaminant should not be ignored.
